# Rho-GTPase pathways may differentiate treatment response to TNF-alpha and IL-17A inhibitors in psoriatic arthritis

**DOI:** 10.1038/s41598-020-78866-2

**Published:** 2020-12-10

**Authors:** Sara Rahmati, Darren D. O’Rielly, Quan Li, Dianne Codner, Amanda Dohey, Kari Jenkins, Igor Jurisica, Dafna D. Gladman, Vinod Chandran, Proton Rahman

**Affiliations:** 1 Krembil Research Institute, UHN, 5-KD405, Krembil Discovery Tower, 60 Leonard Ave, Toronto, M5T 2S8 Canada; 2 Faculty of Medicine, Craig L Dobbin Genetics Research Centre, Memorial University, Suite 3M500, 300 Prince Philip Drive, St. John’s, NL A1B3V6 Canada; 3 University of Toronto, Toronto, Canada; 4 Princess Margaret Cancer Centre, University Health Network, Toronto, ON M5G1L7 Canada; 5 Faculty of Medicine, 5M202 Craig L Dobbin Genetics Research Centre, Memorial University, 300 Prince Philip Drive, St. John’s, NL A1B3V6 Canada; 6 Faculty of Medicine, 5M203 Craig L Dobbin Genetics Research Centre, Memorial University, 300 Prince Philip Drive, St. John’s, NL A1B3V6 Canada; 7 St. Clare’s Mercy Hosptial, 154 LeMarchant Rd., St. John’s, NL A1C5B8 Canada; 8 Data Science Discovery Centre for Chronic Diseases, Krembil Research Institute, Toronto Western Hospital, 60 Leonard Avenue, 5KD-407, Toronto, ON M5T 0S8 Canada; 9 Toronto Western Hospital, 399 Bathurst Street, 1E410B, Toronto, M5T 2S8 Canada; 10 Toronto Western Hospital, 399 Bathurst Street, 1E416, Toronto, M5T 2S8 Canada

**Keywords:** Rheumatology, Rheumatic diseases, Spondyloarthritis, Psoriatic arthritis

## Abstract

Biological therapies have dramatically improved the therapeutic landscape of psoriatic arthritis (PsA); however, 40–50% of patients are primary non-responders with response rates declining significantly with each successive biological therapy. Therefore, there is a pressing need to develop a coherent strategy for effective initial and subsequent selection of biologic agents. We interrogated 40 PsA patients initiating either tumour necrosis factor inhibitors (TNFi) or interleukin-17A inhibitors (17Ai) for active PsA. Patients achieving low disease activity according to the Disease Activity Index for PsA (DAPSA) at 3 months were classified as responders. Baseline and 3-month CD4^+^ transcript profiling were performed, and novel signaling pathways were identified using a multi-omics profiling and integrative computational analysis approach. Using transcriptomic data at initiation of therapy, we identified over 100 differentially expressed genes (DEGs) that differentiated IL-17Ai response from non-response and TNFi response from non-response. Integration of cell-type-specific DEGs with protein–protein interactions and further comprehensive pathway enrichment analysis revealed several pathways. Rho GTPase signaling pathway exhibited a strong signal specific to IL-17Ai response and the genes, *RAC1* and *ROCK*s, are supported by results from prior research. Our detailed network and pathway analyses have identified the rewiring of Rho GTPase pathways as potential markers of response to IL17Ai but not TNFi. These results need further verification.

## Introduction

Psoriasis is a chronic, inflammatory, hyperproliferative skin disease with an estimated prevalence of 2–3%^1^. Approximately 20–30% of psoriasis patients develop a distinctive inflammatory arthritis referred to as psoriatic arthritis (PsA), characterized by enthesitis, synovitis, spondylitis and dactylitis as core musculoskeletal features^2,3^. PsA is a highly heterogeneous entity, with at least one third of PsA patients exhibiting moderate-to-severe disease and thus being managed with biologic disease-modifying anti-rheumatic drugs (bDMARDs) or targeted synthetic disease modifying anti-rheumatic drugs (tsDMARDs)^4^. Clinical parameters alone are not sufficiently characterized to determine the optimal treatment strategy, consequently, a trial-and-error approach represents the norm when initiating advanced therapeutics.

Differential mRNA (gene) expression (DEG) followed by pathway enrichment analysis is a common practice in cell biology and clinical research to study relationship between genetics and phenotypes^5,6^ that has been used in psoriatic disease research too. For example DEGs among psoriasis patients initiating tumour necrosis factor inhibitors (TNFi) and interleukin-17A inhibitors (IL-17Ai) with both lesional and non-lesional skin have been investigated^7,8^. Genes implicated include innate-immune associated genes and those involved in Th-1 and Th-17 response^9,10^. However, these studies do not specifically compare responders versus non-responders in the same class or different class of advanced targeted therapeutics. With respect to PsA, 161 genes were down-regulated, and 27 genes were up-regulated in patients treated with etanercept as compared with untreated controls^11^. Similarly, divergent patterns of altered gene expression in the blood and target organs (i.e., lesional skin and synovial tissue) followed treatment with infliximab in both psoriasis and PsA patients. Enrichment in gene expression related to cell differentiation, proliferation and apoptosis were noted. These function were enriched in CD14^+^ cells in PsA and CD14^+^ and CD14- cells in psoriasis patients^12^. Patients treated with biologic therapy, instituted based on the peripheral blood cell immunophenotype, had significantly greater chance in achieving a good therapeutic response compared to those treated with standard care^13^. However, that study was solely based on cellular phenotyping and thus could not elucidate downstream signalling pathways associated with the differential response.

The primary objectives of this proof-of-concept study were to determine if cell type-specific transcriptomic data obtained at baseline can predict response to biologics at 3 months, and, whether it can help to identify pathways associated with response to biologics. We hypothesize that genomic heterogeneity among PsA patients will lead to differences in response to biologic classes, requiring different treatment regimens. We analyzed CD4^+^ T cell transcriptomes of 40 PsA patients treated with two major classes of biologic agents, TNFi and IL-17Ai, at baseline and at 3 months after treatment and demonstrated that these data can distinguish responder groups. Using an integrative computational systems biology approach, we identified Rho-GTPase pathways and highlighted *RAC1* and *ROCK2* as two important genes in the rewiring of these pathways in different responder groups. Our findings suggest that an intensive, cell-specific pharmacogenomics approach based on bioinformatics and network analysis may represent a solution to the challenge of choosing the right treatment for the right PsA patient. With this approach, critical signaling pathways in PsA are simultaneously illuminated using clinically feasibly collected data.

## Methods

### Clinical

Ethics approval was granted for this study by the Health Research Ethics Authority in Newfoundland and Labrador (2016.195). All patients gave consent to be part of this study and were recruited by the PsA clinic at Memorial University (MUN). All patients satisfied the Classification of Psoriatic Arthritis (CASPAR) criteria^14^. Presence of cutaneous disease was confirmed in all patients, but the extent was not systematically assessed other than the presence of nail involvement. The subtypes of psoriatic arthritis and concomitant use of disease modifying drugs (DMARDs), prednisone, and non-steroidal anti-inflammatory drugs (NSAIDs) are noted below. Consecutive patients initiating TNFi or IL-17Ai therapy were invited to participate in this study. Biologic therapies included any TNFi (golimumab, adalimumab, etanercept, infliximab, and certolizumab pegol) or IL-17Ai (secukinumab and ixekizumab) that were approved for use in Canada at the time of the study. Patients were assessed using a standardized protocol prior to initiation of biologic therapy and 3 months after initiation of therapy. DAPSA is a composite disease activity score for PsA that includes a 68/66 joint count summed with a patient global score, patient pain score, and CRP level. The DAPSA provides a continuous score of arthritis activity and has validated cut-off points for remission (< 4) and low disease activity (< 14). DAPSA disease activity scores have been shown to correlate with functional status and structural progression on radiographs providing further evidence of their validity^15^. As such, responders were defined as patients with DAPSA low disease activity (DAPSA score of less than 14) 3 months after commencing treatment. This was a pragmatic trial, and blood was drawn at the time of clinic visit, and the biologic was started when injection training by a healthcare professional was completed at the patient’s home. The median time from blood collection to starting biologic was 19 days with a mean of 28 days.

### Samples and cell-type specific RNA-seq

EDTA whole blood was collected at two time points per patient: prior to the start of biologics and 3 months after treatment initiation. Peripheral blood mononuclear cells (PBMCs) were separated from whole blood with Ficoll-Paque Plus density gradient (Cat # GE-1440-02, Millipore Sigma), washed, frozen in aliquots and stored in liquid nitrogen. Prior to the start of this study, we isolated CD4^+^ cells and CD14^+^ cells from PsA patients to determine the most discriminatory cell type for this study. From our preliminary analysis, CD4^+^ cells were more discriminatory than CD14^+^ cells for differentiating response to biologic therapy. Consequently, we focused on CD4^+^ T cells for this cohort of 40 patients using the mentioned kit. The isolated beaded cell population was washed and split into two aliquots. DNA was extracted from one aliquot by the traditional salting out technique, and total RNA from the second aliquot with the Lexogen Split RNA Kit (Cat # LEX-008.48, D-Mark Biosciences).

NEBNext Ultra II Directional RNA Library Prep Kit for Illumina (Cat # NEB-E7760L, D-Mark Biosciences) and the NEBNext rRNA Depletion Kit (Cat # NEB_E6310X, D-Mark Biosciences) were used to create sequencing libraries from total RNA. Briefly, total RNA was depleted of ribosomal RNA, enzymatically digested into fragments and reverse transcribed. Double stranded cDNA was purified with Agencourt AMPure XP beads (Cat # A63881, Beckman Coulter). This was followed by end repair and dA-tail addition prior to adaptor ligation. Adaptor-ligated DNA was further purified then unique dual index barcodes attached (NEBNext Dual Indexed Primer Set 1, Cat # E7760D-Mark) and fragments amplified in a PCR reaction. Final libraries were size-selected with bead purification and the quality and quantity assessed with the Tape Station D1000 kit (Agilent) and the KAPA Library Quantification Kit (Cat# KK4828, Roche). Library concentrations were normalized and pooled for sequencing on the Illumina NovaSeq 6000 for 2 × 150 bp reads and ~ 40Million reads/sample.

### Gene expression analysis

Raw FASTQ files with RNA reads were aligned to the human hg19 reference genome using TopHat (v2.1.1)^16^. Quality control steps were performed using FASTQC (Simon Andrews, http://www.bioinformatics.babraham.ac.uk/projects/fastqc/) and RSeqQC. The gene expression for each sample was quantified as Fragments Per Kilobase of transcript per Million mapped reads (FPKM) using Cufflinks (version 2.2.1) and normalized within-sample to TPM (Transcripts Per Million reads) values. All data were transformed as log2(TPM + 1). Batch effects were adjusted using ComBat implemented in the SVA package (version 3.20.0). Differentially expressed genes (DEG) were detected using limma package. Principal Component Analysis (PCA) and plot was performed using R. The heatmaps and hierarchical clustering were generated using the ComplexHeatmap R package^17^. Hierarchical clustering in the heatmap was generated using distance as (1-Pearson correlation) with average linkage.

### Network and pathway analysis

For initial pathway enrichment analysis, given the small sample size, only overlap of DEGs (fold change ≥ 1.4 and raw *p* value ≤ 0.05) between response groups to each biologic at baseline and DEGs between responders to the two biologics were selected. This generated two lists of statistically significant DEGs (hypergeometric test; *p* < 0.008 and 1 × 10^−24^). In order to capture biologically meaningful DEGs, these two gene sets were expanded by overlaying differential expression values onto a physical protein interaction (PPI) network obtained from Integrated Interaction Database (IID, version 2018-11)^18^, with the hypothesis that cell signalling occurs through physical interactions of biomolecules. From the obtained network, gene pairs (fold change ≥ 1.2 regardless of *p* value) whose protein products interact were selected. For pathway enrichment analysis, pathDIP (version 4.1) was used^19^. For detailed Rho-GTPase pathway analysis, union of pathway DEGs (fold change ≥ 1.4 regardless of *p* value) and genes with fold change between 1.2 and 1.4 and a raw p-value of ≤ 0.05 were used. Network Analysis, Visualization, and Graphing TORonto (NAViGaTOR^20^, version 3) was used to visualize networks. We used wordle.net (as of June 2020) to build wordclouds. The R package gplots (version 3.0.1.1) was used to generate heatmaps while lists of “Rho family GTPases (Rho)” and “Rho-GTPase activating Proteins (ARHGAP)” were obtained from Human Gene Nomenclature Committee (HGNC), one of the committees of the Human Genome Organization (HUGO) (version April 2020). Focusing on genes involved in Rho-GTPases and their related pathways, particularly with respect to expression at baseline, as well as between pre- and post-treatment, seven comparisons were analyzed in greater detail: 1) responders and non-responders to TNFi or IL-17Ai at baseline (two comparisons); 2) responders to TNFi and IL-17Ai at baseline (two comparisons); 3) responders to TNFi or IL-17Ai at baseline and after 3 months (two comparisons); and 4) non-responders to TNFi or IL-17Ai at baseline and after 3 months (one comparison). We obtained *ROCK2* and *RAC1* protein expression data across 19 different tissues from the Human Protein Atlas^21^. We used mRNA expression data of these two genes provided by GTEx consortium^22^ and processed by the Human Protein Atlas (October 2020). We used Pearson correlation to calculate correlation (R) between mRNA and protein levels.

## Results

**Figure 1:**
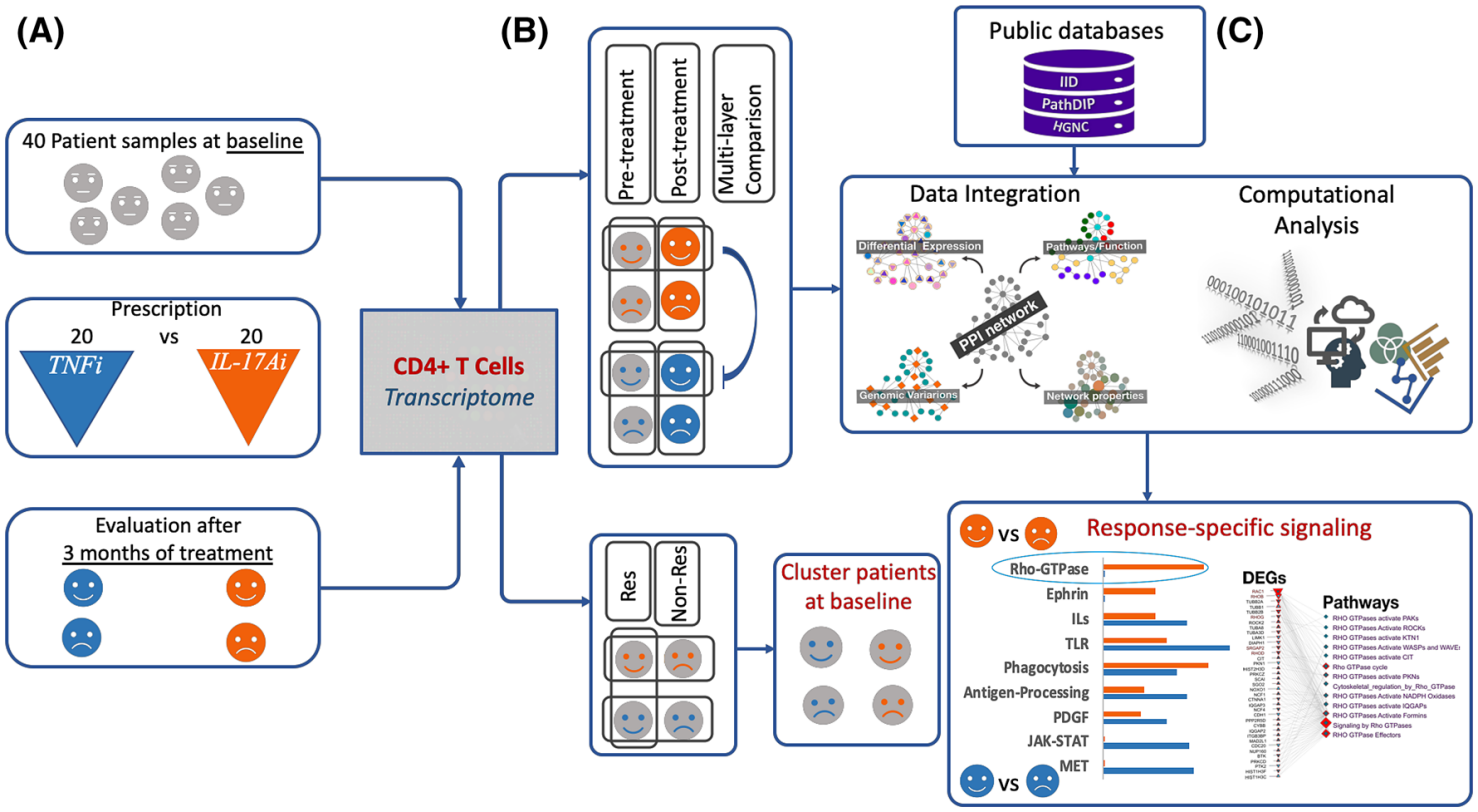
Schematic overview of study. (**A**) CD4^+^ T-cell transcriptomes of 40 patients with active PsA, at baseline and 3 months after treatment with TNFi or IL-17Ai. (**B**) Hierarchical clustering and PCA analysis clearly distinguished response groups. (**C**) Since the protein products of deregulated genes perform in groups, their physical connectivity can bring an additional layer of confidence to selection of genes whose statistical significance may not reflect their importance due to small sample size. We developed a multilayer analysis approach that annotates protein interactions with DEGs, protein families, and pathways. Our analysis identified signaling pathways likely involved in response to each biologic.

Figure 1 illustrates the outline of this study, and Table 1 shows demographic and disease characteristics of the study subjects. There were 13 responders (65%) and seven non-responders (35%) in the TNFi group with biologic treatment naïve (bio-naïve) and previously-exposed (bio-exposed) patients exhibiting a 66% (8/12) and a 37.5% (3/8) response rate, respectively. There were seven responders (35%) and thirteen non-responders (65%) in the IL-17Ai group with bio-naïve and bio-exposed patients exhibiting a 50% (4/8) and a 25% (3/12) response rate, respectively. Low DAPSA disease activity was obtained for 50% (4/8) and 25% (3/12) of bio-naïve and bio-exposed patients, respectively.

**Table 1:** Demographic and disease characteristics of patients in study.

	IL-17Ai	TNFi
Number of PsA patients	20	20
Sex (% female)	55%	75%
Mean Age (S.D.) in years	55.9 (9.5)	56.8 (7.9)
Mean disease duration (S.D.) in years	10.4 (6.9)	7.3 (7.9)
Polyarticular PsA	100%	100%
Axial involvement	65%	65%
Nail involvement	50%	40%
Mean DAPSA (S.D.) baseline	38.8 (17.5)	45.6 (28.9)
Responders at 3 months (%) (DAPSA < 14)	35%	60%
NSAIDs	35%	40%
Prednisone	10%	25%
DMARDs	30%	35%
Biologic treatment naïve (%)	40%	60%

*IL17Ai* IL17A inhibitors; *TNFi* TNF inhibitors; *DAPSA* Disease Activity Index for Psoriatic Arthritis.

**Figure 2:**
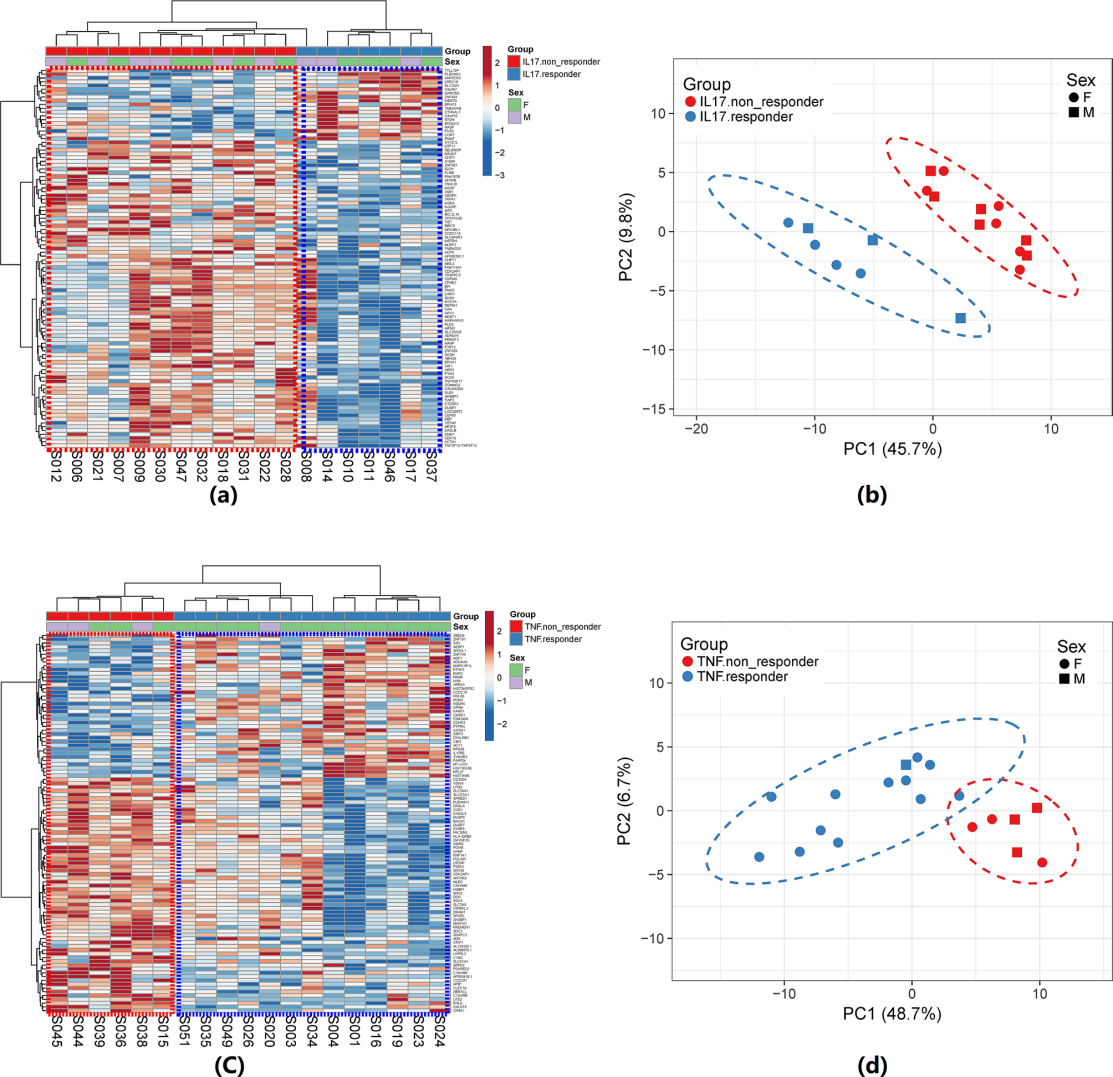
Sample Clustering and PCA analysis based on CD4^+^ cell transcriptomic data. The hierarchical clustering in heatmaps and 2D PCA plots with ellipses concentration clearly show separation of the responder and non-responders. In the heatmaps, columns are samples and rows are differentially expressed genes, the expression levels are presented as median-centered. Samples in the red-dashed lines were non-responders and samples in the blue-dashed lines were responders. (**a**) and (**b**) are heatmap and PCA plot for IL17i responder and non-responders; (**c**) and (**d**) are heatmap and PCA plot for TNFi responder and non-responders.

**Figure 3:**
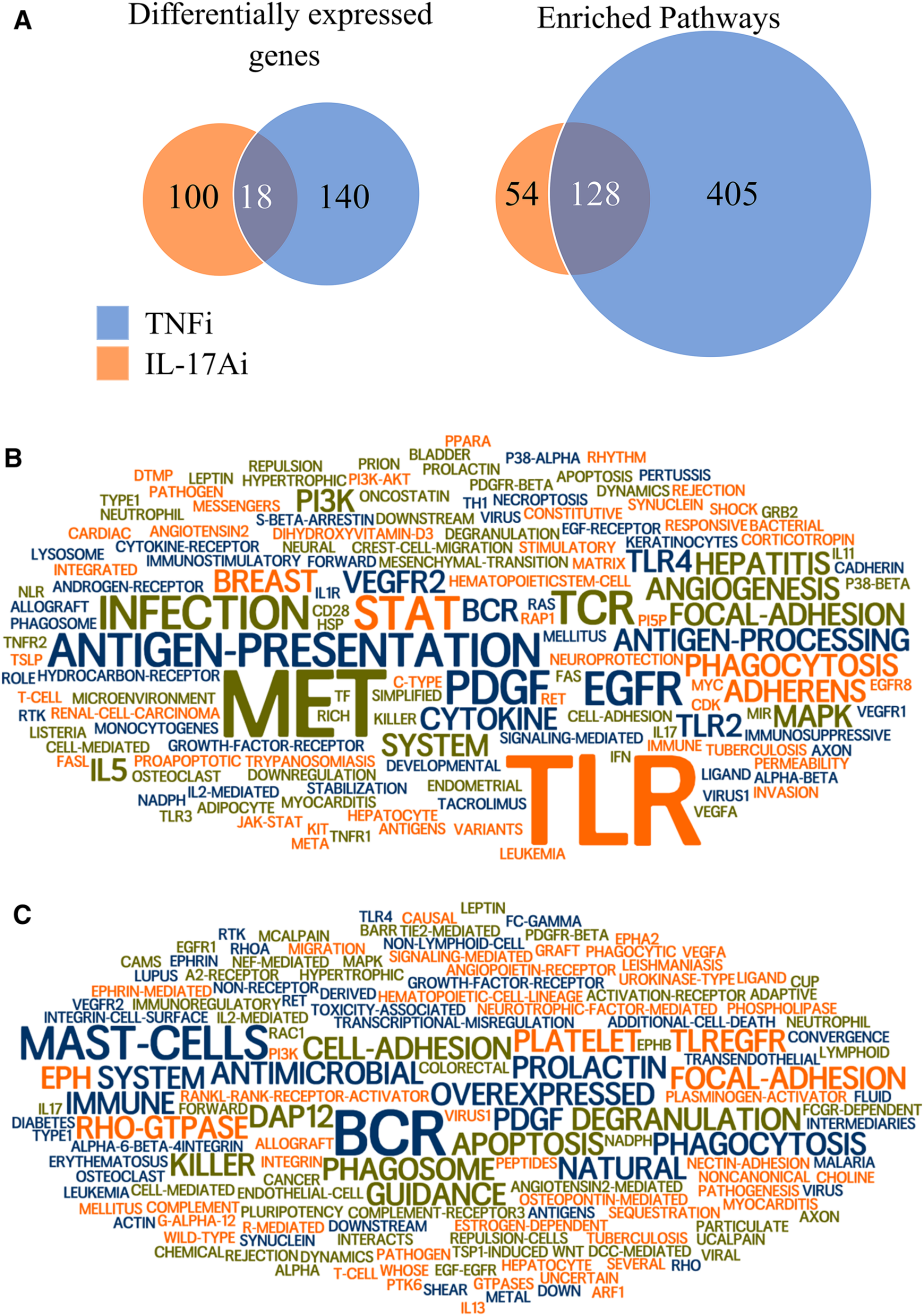
Pathway enrichment analysis of selected DEGs using PPI network. (**A**) Overlap of DEGs between response groups to each biologic at baseline and their enriched pathways. (**B**,**C**) Key-terms of pathways enriched in DEGs between response groups to (**B**) TNFi and (**C**) IL-17Ai. Size of the terms is proportional with statistical significance of each term in titles of enriched pathways. Colors are different only for clarity. While most of the large terms in each panel are present in the other panel in a different size, MET, JAK, STAT in panel B, and, Rho-GTPase and EPH in panel C are absent from the other panel. Thus, high deregulation of these pathways between responders and non-responders is specific to one of the two biologics.

Clustering and PCA of samples based on the top 100 best *p* values DEGs (|FC|> = 1.4, *p* < 0.05) revealed a clear distinction between responders and non-responders for each group of patients, and between response and non-response to each of the two biologics (Fig. 2). The results from the PCA that the cluster of responders is distinct compared with non-responders was confirmed by the heatmap and hierarchical clustering. However, pathway analysis of these genes failed to identify enriched pathways. Furthermore, lists of DEGs across three comparisons before treatment (i.e., response groups to each of IL-17Ai and TNFi and responders to the two biologics), share only two genes (*ARMCX2; CRIM1*) (Supplementary Table 1). Although this low overlap is statistically significant (randomization test, *p* < 0.001), this could be attributed to the small number of samples, thus missing potentially overlapping signals due to low statistical power for DEG analysis. To overcome this potential problem, a multi-layer integrative computational heuristic approach was used to distinguish functionally related groups of DEGs that may be distinctive of responders and non-responders to each biologic from noise. Highly DEGs between responders and non-responders to IL-17Ai (26 genes) and between responders and non-responders to TNFi (6 genes) were selected (Supplementary Table 2). Based on the hypothesis that using PPIs can compensate for low statistical power of our analysis, the two lists were expanded, revealing 118 and 158 DEGs between responders and non-responders to TNFi and IL-17Ai, respectively (Supplementary Table 3). Comprehensive pathway enrichment analysis of these genes revealed 534 (out of 5380) pathways enriched for 158 DEGs between responders and non-responders to TNFi, and 182 pathways enriched for 118 DEGs between responders and non-responders to IL-17Ai at baseline (Fig. 3A, Supplementary Tables 4–5).

Key-terms of pathways in both groups are illustrated (Fig. 3B,C). Rho-GTPase pathways were selected for further detailed analysis since it provides the strongest evidence among the differential pathways. Several pathways tightly related to Rho-GTPase pathways such as EPH/Ephrin-related pathways and PTK6 are specific to the IL-17Ai group. Analysis of DEG annotations revealed that out of sixteen Rho-GTPase pathways present in pathDIP, thirteen have at least one DEG in the IL-17Ai group, while only six are present in the TNFi group. Among enriched pathways in DEGs in the IL-17Ai group, Rho-GTPase pathway has the highest number of DEGs (Supplementary Tables 6–9).

**Figure 4:**
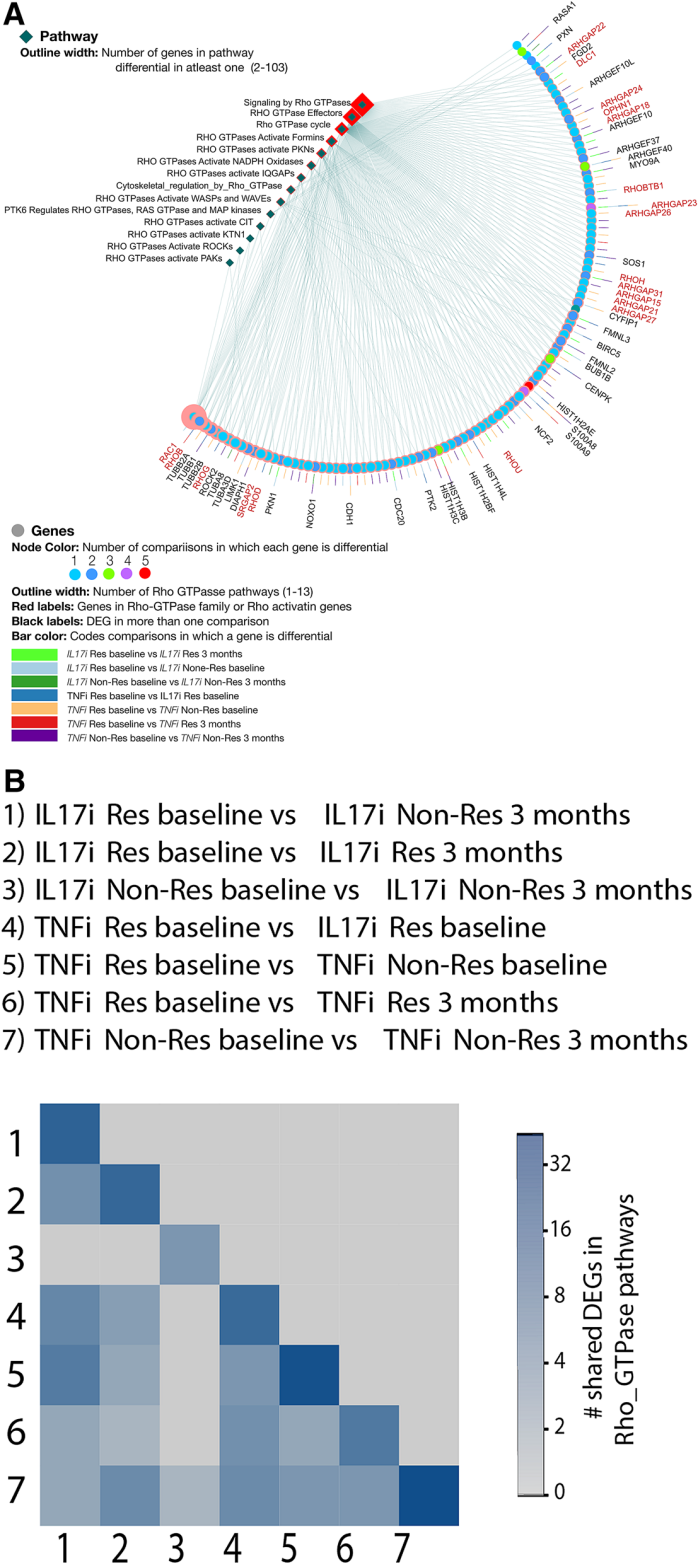
DEGs in Rho-GTPase pathways and their overlap. (**A**) Membership of DEGs in Rho-GTPase pathways. Black labels show DEGs in more than one comparison or members of more than 4 Rho-GTPase pathways. Red labels show DEGs whose protein product is characterized as Rho family GTPase or Rho-GTPase activating protein (based on HGNC annotation). (**B**) Overlap size of Rho-GTPase DEGs in pairs of comparisons.

**Figure 5:**
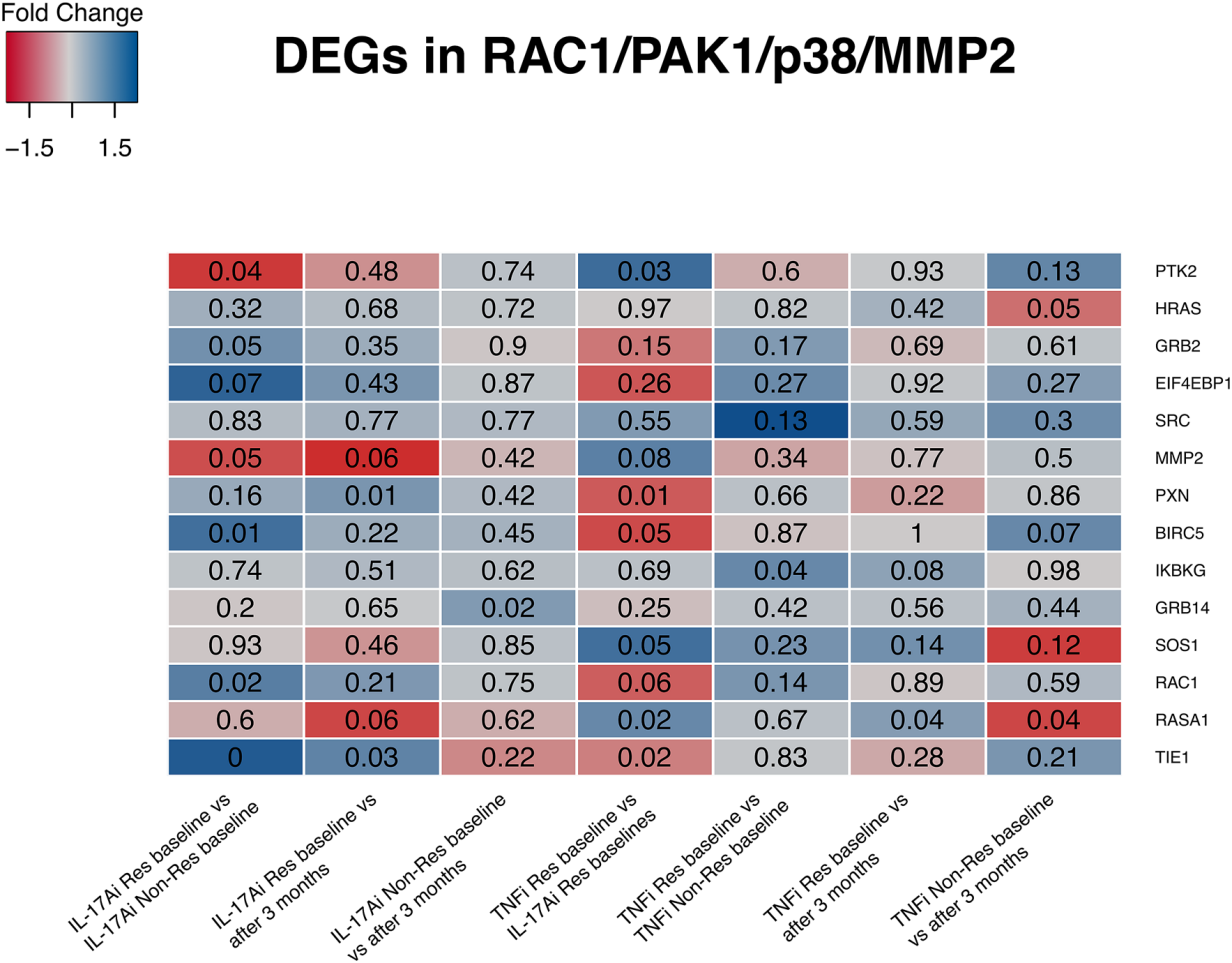
Fold change (represented with color) and *p* value (shown in numbers in the cells) of DEGs in RAC1/PAK1/p38/MMP2 pathway across different comparisons. Pathway genes were downloaded from PathDIP. Each column represents DEGs between two groups of “group1” and “group 2” and “group1 versus group2” comparison, fold change shows expression in group 1 minus expression in group 2. The range of fold change changes between negative 1.5 (downregulated in group 1) and positive 1.7 (upregulated in group 1). Color-code shows log2 of fold change values. The two columns that compare IL17Ai responder group to IL17Ai non-responders and TNFi responders pre-treatment show the highest number of significant pathway DEGs (6 out of 14). In addition, fold change of RAC1 is statistically significant only in IL17A-i responders versus IL17Ai non-responders pre-treatment, and its *p* value (0.06) is only slightly above the threshold of 0.05 in IL17Ai responders versus TNFi responders pre-treatment.

Using the keyword “RHO-GTPase”, sixteen pathways from pathDIP were retrieved which included 485 unique proteins, with fourteen pathways containing at least one DEG and all together covering 108 DEGs in at least one comparison (Fig. 4A). *RAC1* appears in 13/14 Rho-GTPase pathways with the “RAC1/PAK1/p38/MMP2” pathway enriched in DEGs between responders versus non-responders to IL-17Ai, and responders to IL-17Ai versus responders to TNFi at baseline, but not between responders versus non-responders to TNFi. “RAC1/PAK1/p38/MMP2” is a pathway with 68 members of which seven are DEGs in responders versus non-responders to IL-17Ai and five are DEGs between responders to TNFi versus IL-17Ai at baseline. Interestingly, *RAC1* is detected as a DEG only between responders and non-responders to IL-17Ai at baseline. As expected, responders to the two biologics at baseline revealed the highest overlap with the other comparisons (Fig. 4B). The “RAC1/PAK1/p38/MMP2” pathway was enriched in DEGs between responders versus non-responders to IL-17Ai, and responders to IL-17Ai versus responders to TNFi at baseline, but not between responders versus non-responders to TNFi (Fig. 5). Interestingly, *RAC1* is detected as a DEG only between responders and non-responders to IL-17Ai at baseline, which is consistent with a trend observed with RAC1 fold change and *p* value in DEG analysis of responders to the two biologics at baseline (Fig. 5).

## Discussion

In this study, over 100 DEGs were identified that differentiated IL-17Ai response from non-response and TNFi response from non-response at initiation of therapy. Integration of cell-type-specific DEGs with PPIs and further comprehensive pathway enrichment analysis revealed 534 pathways enriched between responders and non-responders to TNFi, and 182 pathways enriched between responders and non-responders to IL-17Ai. Interestingly, while these two gene lists share only 18 genes, they have 128 enriched pathways in common suggesting a potential effect of two different treatments on similar pathways but through different pathway members. Moreover, it suggests potential importance of the 18 shared genes enriched in 19 immune pathways (Supplemental Table 10) in association with these pathways. The majority of these 19 pathways are related to innate and adaptive immune system signaling and immune-related diseases.

Interestingly, we found several signaling pathways specific to either the TNFi group or the IL-17Ai group. JAK and STAT are among the most highlighted pathways specific to the TNFi group. Identifying JAK/STAT pathway is interesting as *JAK* inhibitors are a major class of therapies that have been effective in non-responders to TNFi in PsA patients^23^. Although enrichment results alone show strong signaling toward specificity of the JAK pathway to the TNFi group at baseline, evidence for its specificity is not as strong as evidence for specificity of the Rho-GTPase pathway to the IL-17Ai group at baseline. Specificity of Rho-GTPase pathway is supported by specificity of EPH/Ephrin-related pathways and the PTK6 signaling pathway to IL-17Ai response but not to TNFi response. Interestingly, EPH/Ephrin-related pathways are tightly related to Rho-GTPase proteins *RAC1*, *RHOA*, and CDC42^24–26^, while *PTK6* is a regulator of *RAC1* and *RHOA* activity^27,28^. Annotating genes in sixteen available Rho-GTPase pathways with DEG values across seven comparisons, and focusing only on membership of DEGs in pathways regardless of their enrichment, revealed 14/16 Rho-GTPase pathways included at least one DEG.

Non-responders to IL-17Ai before and after treatment have only four DEGs in Rho-GTPase pathways, suggesting low effect of IL-17Ai on Rho-GTPase pathways in non-responders. In contrast, non-responders to TNFi include a high number of DEGs in these pathways (48/108) followed by responders versus non-responders to each biologic before and after treatment. Interestingly, 70/108 genes are DEGs in only one comparison. The two comparisons that involved non-responders to TNFi, cover the highest number of these 70 genes (26 and 23), suggesting the potential of Rho-GTPase pathways in distinguishing TNFi non-responders from other groups. As expected, responders to the two biologics at baseline revealed the highest overlap with the other comparisons, suggesting Rho-GTPase pathways as good candidates not only in stratifying PsA patients into responders and non-responders to different biologics, but also their possible contribution in understanding the mechanism of action of IL-17Ai and TNFi in PsA.

Our network-based approach suggests that *RAC1* in PsA patients may represent a potential predictor of treatment response for IL-17Ai. *RAC1* is a Rho-GTPase which acts as a “molecular switch”, known for its role in several cellular processes such as cytoskeleton organization, gene expression regulation, cellular plasticity, production of reactive oxygen species, cellular adhesions, migration and invasion, cell proliferation, apoptosis, and inflammatory responses^29–36^. *Rac1* appears to be active in multiple cell types involved in psoriatic disease, including epithelial cells^37^, fibroblasts^37^, macrophages^38^, T cells^39^ and osteoclasts^40^. Evidence from genome-wide association studies in psoriatic disease supports an important role of *Rac1* in psoriatic disease. Several genetic association studies of psoriatic disease have implicated genes described previously to interact with Rac1, such as *ZNF750, STAT3, NFkB, ELMO1, NOS2, IRFs* and B*-defensins*^41–46^. *RAC1* is a key orchestrator of pathologic epidermis-immune interaction, which is of particular interest in psoriatic disease. There is a marked activation of Rac1 in psoriatic lesional epidermis compared with normal control skin^47^, and overexpressing a *Rac1V12* mutant under a keratin 14 promoter in a transgenic mouse model recapitulated many hallmarks of human psoriasis, including psoriasiform hyperplasia, the Auspitz sign, Koebnerization, joint inflammation and a mutilating arthropathy^29^. Importantly, transgenic Rac1 mice demonstrate the Auspitz sign and Koebnerization phenomenon, as well as epidermal *RAC1* hyperactivation being sufficient to promote disease activity in the skin, nails, and joints which closely mimics human psoriasis^29^. In keratinocytes, modulating *RAC1* activity altered differentiation, proliferation, and inflammatory pathways, including *STAT3*, *NFκB*, and ZINC finger protein 750 (*ZNF750*)^29^. *Rac1* plays an important role in antigen presentation and bone remodelling^40,48,49^. Perturbed antigen presentation has been implicated in the pathogenesis of psoriatic disease, and *Rac1* has been demonstrated to play a role in antigen-presentation in dendritic cells, at least in part through increased endocytosis^48^. Engagement of the T cell receptor, secondary to antigen presentation, elicits a complex cascade of signalling events that activate *Rac1*, which forms a dynamic structure in close contact with the antigen presenting cell^49^. These findings implicate *RAC1* as a potential therapeutic target for psoriatic disease, supporting our results.

In addition, the only Rho-GTPase pathway that does not include *RAC1* is “Rho-GTPases activate *ROCK*s” pathway. This finding is intriguing since it has been shown that experimental inhibition of *ROCK2*, a target of Rho-GTPase family, is effective in psoriatic disease through regulation of *IL-17/23/10*, but not *IL-6* and *TNFα*^50^. According to our data, although *ROCK2* expression is not differentially expressed between responders and non-responders to each biologic at baseline, its expression is noticeably changed before and after treatment in responders to IL-17Ai and TNFi, as well as in non-responders to TNFi, but not in non-responders to IL-17Ai. Collectively, these data suggest a complex interplay among three genes (*IL-17A*, *TNF*, and *ROCK2*) in PsA pathogenesis and treatment response.

Importantly, although in our initial pathway enrichment analysis we found several Rho-GTPase-related pathways enriched only in DEGs at baseline between responders and non-responders to IL-17i, detailed network analysis (investigating DEG membership in pathways regardless of enrichment) implicated these pathways in response to both biologics. In fact, there is one Rho-GTPase pathway, “RHO-GTPases Activate NADPH Oxidases”, which is enriched in DEGs between response groups to both biologics at baseline. NADPH oxidase is a protein complex that, interestingly, is found in the membrane of phagosomes and engulfs microorganisms in neutrophil^51^. The importance of this protein complex in psoriatic disease and response of psoriatic patients to therapy has been studied for over 30 years^52^, further supporting our integrative method of capturing relevant signals from background noise.

This study suggests that analysis of cell-type-specific transcriptomic data of PsA patients at baseline is a promising approach to cluster responder groups to biologic treatments. However, there are some limitations to the study. Given that PsA is a T cell-mediated disease, which encompasses Th-1, as well as Th-17, Th-22, Th-9 and T-reg subsets^53^, limiting gene expression and pathway analyses to CD4^+^ cells represents a limitation. Our analyses show significant differences in mRNA expression of producer genes in Rho-GTPase pathways and do not prove differences in their protein levels. However, previous studies have reported the effect of inhibition of protein and mRNA expression of Rho-GTPases (including *ROCK2*) with the activity of their downstream pathways, and, with mRNA and protein levels of *IL-17*^54–56^. For example, it has been shown that inhibition of *ROCK* proteins results in decreased activation and elevated apoptosis of CD4^+^ T-cells, suppressed expression of *IL-17* and IL-4, and inhibition of differentiation and secretion of Th-17 cells in mice^56^. Furthermore, correlation between mRNA with protein and activity levels of *ROCK2* captured through small-screen experiments has been reported in different tissues, diseases, and cell lines^57,58^. To further support correlation between mRNA expression and protein levels of *ROCK2* we compared its protein and mRNA expression across 19 different tissues and found a statistically significant correlation between them (R = 0.53 and *p* = 0.01) (Supplemental Table 11). Similar data is provided for *RAC1* (R = 0.54, *p* = 0.01) (Supplemental Table 12); note that our analyses identified *RAC1* as a potential prognostic marker of PsA patients in response to different biologic therapies. Despite this evidence, further functional validation of our findings is necessary. It is conceivable that our results could be confounded by previous treatment with DMARDs and biologic agents^59,60^, which will be addressed in future studies with larger number of patients as sub-setting patients into bio-naïve and bio-exposed would have resulted in very small sample sizes. Furthermore, the use of biologics was not randomized, the assessors were not blinded to assess the clinical response, and there is subjectivity in determining responder status. Another potential confounder was the previous use of therapy in some patients. Sub-setting patients into bio-naïve and bio-exposed would have resulted in very small sample sizes. While combining these samples afforded capture of signals shared across subsets, stratifying patients to previously exposed versus naïve to biologics may help detect subset-specific signals. In addition, further studies are necessary to understand whether part of apparently relevant, but missing, signals in our analysis in IL-17Ai group are due to the small number and heterogeneity of sample groups. Examples of such signals are pathways including Th-1, Th-2, and Th-17 cell differentiation, which were enriched in DEGs between responders and non-responders to TNFi at baseline, but not in IL-17Ai group. Missing such pathways may also be partly related to inconsistency in ontologies and nomenclatures used by different source pathway databases in pathDIP. For example, while KEGG^61^ includes pathways specific to T-cell helpers, Reactome^62^ groups them only as part of Interleukin pathways. Moreover, integrating other types of biological data such as cellular localization, genomic aberrations, and annotation with previously known PsA genes, may help improve results. Nevertheless, our cell-type-specific integrative network-based approach proved successful in capturing strong trends consistent with previous studies in humans and mice and represents a promising approach for data-driven hypothesis generation. Importantly, our method is applicable to data whose collection is practical from a clinical point of view. The next phase of this work will involve larger cohorts to account for more co-variates and balance the differences in baseline features of the treatment groups.

In summary, this study suggests analysis of cell-type-specific transcriptomic data of PsA patients at baseline is a promising approach to cluster responder groups to biologic treatments with potential to be used as a systematic approach to help clinicians in determining optimal treatment. Importantly, integration of these data with data available in public databases such as PPI networks and pathways improved distinguishing biologically relevant signals from noise. We identified several pathways specific to DEGs between responders and non-responders to different biologics. In particular, we identified Rho-GTPase pathways as good candidates to distinguish differences between responders and non-responders to IL-17Ai. Detection of these relevant signals was possible with data integration and network analysis given small sample size and high heterogeneity between subjects.

## Conclusions

Integration of CD4^+^ T cell-specific transcriptomic data with PPI networks, pathways, and protein families represents a very promising strategy for predicting treatment responders. In addition, it can detect pathways and genes involved in response to biologics. Using this approach, we identified Rho-GTPase pathway and its members such as *RAC1* and *ROCK*s as potential markers to guide the choice of biologic agents for individual PsA patients and study signaling cascades in PsA.

## Supplementary Information

Supplementary Information
